# Alpha-1-Antitrypsin Ameliorates Pristane Induced Diffuse Alveolar Hemorrhage in Mice

**DOI:** 10.3390/jcm8091341

**Published:** 2019-08-29

**Authors:** Ahmed S. Elshikha, Georges Abboud, Lonneke van der Meijden-Erkelens, Yuanqing Lu, Mong-Jen Chen, Ye Yuan, Godelieva Ponjee, Leilani Zeumer, Minoru Satoh, Laurence Morel, Sihong Song

**Affiliations:** 1 Department of Pharmaceutics, College of Pharmacy, University of Florida, Gainesville, FL 32610, USA; 2 Department of Pharmaceutics, Zagazig University, Zagazig, Sharkia 44519, Egypt; 3 Department of Pathology, Immunology and Laboratory Medicine, University of Florida, Gainesville, FL 32610, USA; 4 Department of Clinical Nursing, University of Occupational and Environmental Health, Kitakyushu 807-8555, Fukuoka, Japan

**Keywords:** Lupus, Diffuse alveolar hemorrhage (DAH), alpha-1-antitrypsin (AAT)

## Abstract

Diffuse alveolar hemorrhage (DAH) is a fatal complication in patients with lupus. DAH can be induced in B6 mice by an intraperitoneal injection of pristane. Since human alpha-1-antitrypsin (hAAT) is an anti-inflammatory and immuno-regulatory protein, we investigated the protective effect of hAAT against pristane-induced DAH in B6 mice and hAAT transgenic (hAAT-Tg) mice. We first showed that hAAT Tg expression lowers TNF-α production in B cells, as well as CD4+ T cells in untreated mice. Conversely, the frequency of regulatory CD4+CD25+ and CD4+CD25-IL-10+ cells was significantly higher in hAAT-Tg than in B6 mice. This confirmed the anti-inflammatory effect of hAAT that was observed even at steady state. One week after a pristane injection, the frequency of peritoneal Ly6C^hi^ inflammatory monocytes and neutrophils in hAAT-Tg mice was significantly lower than that in B6 mice. Importantly, pristane-induced DAH was completely prevented in hAAT-Tg mice and this was associated with a modulation of anti- to pro-inflammatory myeloid cell ratio/balance. We also showed that treatment with hAAT decreased the severity of DAH in B6 mice. These results showed for the first time that hAAT has a therapeutic potential for the treatment of DAH.

## 1. Introduction

Systemic lupus erythematosus (SLE) is a chronic autoimmune disease that is characterized by the overexpression of autoantibodies causing multiple organ damage due to loss of tolerance to self-antigens [1]. Lung disease occurs in half of SLE patients [2], in which diffuse alveolar hemorrhage (DAH) is a rare, but serious, complication of SLE [3,4]. Its prevalence ranges from 1–5% and leads to >50% mortality [3,5,6,7,8]. DAH is characterized by capillaritis, hemorrhage, and interstitial infiltration by mononuclear and polynuclear leukocytes, alveolar necrosis, and deposits of hemosiderin macrophages [7,9,10]. Current treatments for DAH include steroids alone or in combination with immunosuppressive drugs, plasmapheresis, and mechanical ventilation [3,11]. However, DAH can often recur and is usually fatal [6,12]. Therefore, there is an unmet need for a safe and effective treatment of DAH. 

Pristane (2, 6, 10, 14-tetramethylpentadecane) is a hydrocarbon oil that can induce SLE-like syndrome with lupus specific autoantibodies, nephritis, arthritis, and DAH after a single intraperitoneal (IP) injection in C57BL/6 (B6) mice [2,3,8,13,14,15]. The ability of pristane to induce type I interferons (IFN-I) [13,16] and alveolar hemorrhage in B6 mice is unique, making it the only animal model of lupus-associated DAH [15,17]. Previous studies have reported that within 2 weeks of pristane administration, accumulation of peritoneal exudate cells (PECs), particularly Ly6C^hi^ monocytes, Ly6C^lo^ monocytes/macrophages, and Ly6G^+^ neutrophils can be detected in the peritoneum [7,13,14]. Ly6C^hi^ monocytes are the major source of IFN-I in this model and their recruitment and accumulation in peritoneum persists for as long as 4 months after pristane treatment [18]. In addition, these inflammatory Ly6C^hi^ monocytes rapidly turnover after pristane injection and do not differentiate into the anti-inflammatory Ly6C^lo^ monocytes/macrophages [16]. Recent studies have highlighted the detrimental role of neutrophil extracellular trap (NET) in pristane-induced DAH [2,8]. TNF-α is a potent inflammatory cytokine that is one of the earliest mediators produced in response to several inflammatory stimuli [19]. It has been implicated in a variety of pulmonary diseases, including asthma [20], chronic obstructive pulmonary disease (COPD) [21], emphysema [22], and pulmonary fibrosis [23]. TNF-α levels are elevated in pristane-treated mice and lead to bone marrow dysfunction, dyserythropoiesis, and anemia [24]. Importantly, pristane-treated TNFα^−/−^ mice do not develop hematological abnormalities [24]. Moreover, pristane induced arthritis was ameliorated with TNF-α inhibitor therapy, indicating its critical role in pristane-induced pathogenesis [25]. In addition to TNF-α, other inflammatory cytokines, such as IL-1β, IL-6, and IL-10 are found in pristane-treated mice [26]. Collectively, these studies indicated the pathogenic role of innate immune response stimulation in pristane induced DAH.

Serine protease inhibitors (SERPIN) are immune regulators for multiple pathways and are present in all kingdoms of life, including vertebrates, invertebrates, plants, viruses, and bacteria [27]. In mammals and humans, SERPINs are major serum proteins, and play important roles in clotting and inflammatory pathways [28]. Some viruses use SERPIN to modulate host immune responses for their survival and functions. For example, myxoma viruses express three SERPINs, SERP1, SERP2, and SERP3 [29], and orthopox viruses express three SERPINs, SPI-1, SPI-2, and SPI-3 [30]. In fact, some of the viral SERPINs have been developed as a drug for therapeutic applications [31,32,33]. It has been shown that SERP1 can inhibit monocyte function [34] and suppress collagen-induced arthritis (CIA) [35]. SERP1 treatment can reduce inflammatory macrophage invitation and cancer cell growth [36]. Interestingly, SERP1 treatment can also reduce virus load and lung hemorrhage, as well as aortic, lung, and colon inflammation in murine gammaherpesvirus 68 (MHV68)-infected mice [37]. Clinical studies using SERP1 to treat acute coronary syndromes (ACS) have shown promising results [38]. Increasing evidence showed that SERPINs has therapeutic potential for inflammatory diseases. Human alpha-1-antitrypsin (hAAT) is a SERPIN mainly synthesized by liver, and its well-known function is to inhibit neutrophil elastase and prevent emphysema [39,40,41]. Similar to some of the viral SERPINs, increasing evidence has shown that hAAT is a multifunctional protein with anti-inflammatory, cytoprotective, and immunoregulatory properties [41,42,43,44,45]. Specifically, hAAT can inhibit TNF-α expression [46]. Human alpha-1-antitrypsin (hAAT) can also interact with TNF-α receptors and block TNF-α signaling [47]. AAT deficiency (AATD) leads to increased TNF-α signaling and excessive neutrophil degranulation. Conversely, treatment of AATD patients with AAT augmentation therapy decreases neutrophil membrane TNF-α expression and decreases plasma levels of granule antigenic proteins [46]. We have shown that hAAT inhibits the activation of murine bone marrow derived dendritic cells (BMDCs) by lipopolysaccharides (LPS) or CpG, a TLR4 and TLR9 agonist, respectively, and their secretion of cytokines such as TNF-α, IL-6, IL-12, IL-1β, and IFN-I [48,49]. Moreover, hAAT protein and gene therapy inhibited the production of autoantibody and ameliorated outcomes in spontaneous lupus mouse models [48].

Since DAH is a complication of SLE, and hAAT has a therapeutic potential for treatment of SLE, we tested in this study the protective effect of hAAT against pristane-induced DAH. We employed hAAT protein therapy, hAAT-Tg mice, and mouse-AAT knockout mice on a B6 genetic background. We showed an immunomodulatory effect of hAAT on immune cell activation, and importantly showed a significant reduction of DAH by hAAT provided either by transgenic expression or protein treatment.

## 2. Materials and Methods

### 2.1. Animals

B6 female mice were purchased at 8–12 weeks old from Jackson Laboratories (Bar Harbor, Maine). Mouse-AAT knock out (AAT-Ko) mice were generously provided by Dr. Christian Mueller, University of Massachusetts [50], and bred at the University of Florida. The hAAT-Tg mice were developed and maintained at the University of Florida. They were initially generated on NOD background using a hAAT expression cassette (hAAT cDNA, GeneBank M11465.1, driven by the Cytomegalovirus (CMV) enhancer and the chicken beta-actin promoter) flanked with AAV2 inverted terminal repeat sequences (ITRs, rAAV2-CB-hAAT) and crossbreed with B6 mice [51]. All mice were housed in specific pathogen-free (SPF) conditions.

For induction of DAH, 10- to 12-week-old hAAT-Tg, B6, and AAT-Ko mice were randomly assigned into control or treated groups. Control mice were treated with one 0.5 mL Intraperitoneal (IP) injection of phosphate-buffered saline (PBS) (Corning Cellgro, Manassas, VA, USA). Pristane-treated mice received one 0.5 mL IP injection of pristane (Sigma-Aldrich, St. Louis, MO, USA). For experiments testing hAAT as a protein therapy, mice were injected with a clinical grade of hAAT (Prolastin C^®^, Grifols, NC, USA; 2 mg/mouse in 100 µL PBS, every two days) starting one week before pristane injection and continued until the end of the experiment. A total of 53 B6, 6 AAT-Ko, and 8 hAAT-Tg mice were used in this study. The experimental groups are listed in Table S1. All experiments were conducted according to protocols approved by the institutional animal care and use committee (IACUC) (UF-IACUC number: 201907848) at the University of Florida.

### 2.2. Splenocyte Stimulation

Spleens were disrupted with two frosted microscope slides (Fisher Scientific, Pittsburgh, PA, USA) in cold RPMI 1640 (Corning Cellgro, Manassas, VA, USA) containing 10% fetal bovine serum (FBS) (Thermo Scientific, Pittsburg, PA, USA), 1% Penicillin-Streptomycin (Corning Cellgro, Manassas, VA, USA), and 2-Mercaptoethanol (Sigma-Aldrich, St. Louis, MO, USA), and then passed through 70 µm filters (Thermo Scientific, Pittsburg, PA, USA). The cell suspension was then treated with red blood cell (RBC) lysis solution containing 1M Tris, NH_4_CL, pH 7.5 for 5 min at room temperature. Then cells were washed 2 times with RPMI 1640 medium and resuspended in the same medium. Sterile 12 or 24 well microplates (Costar, St. Louis, MO, USA) were used to culture the cells at density of 1 × 10^6^ per well. Plates were incubated at 37 °C/5% CO_2_. 

For splenocyte stimulation, we used leucocyte activation cocktail (LAC, BD Bioscience, San Diego, CA, USA), which is a ready-to-use polyclonal cell activation mixture containing the phorbol ester, PMA (Phorbol 12-Myristate 13-Acetate), a calcium ionophore (Ionomycin), and the protein transport inhibitor BD GolgiPlug^™^ (Brefeldin A). Some splenocytes were stimulated with 1 µg/mL LAC for 6 h, then cells were stained and analyzed by flow cytometry. For the detection of cytokine production, cells were stimulated with either 10 µg/mL LPS or 1 µg/mL Resiquimod (R848) (Invivogen, San Diego, CA, USA) as TLR7/8 agonist for 6, 12, 24, and 48 h. After each time point, cells were centrifuged, and the supernatant was stored at −80 °C for AAT and cytokine detection by ELISA.

### 2.3. Flow Cytometry

Peritoneal exudate cells or splenocytes (1 × 10^6^ cells) were blocked on ice with Fc receptor blocker, anti-CD16/CD32, (93). Then cells were stained with the following FITC-, PE-, PECy7-, PB-, BV421-, APC-, PerCP, monoclonal antibodies to mouse CD11b (M1/70), CD11c (HL3), PDCA-1 (927), I-A/I-E (M5/114.15.2), CD4 (RM4-5), CD3 (145-2C11), CD8 (53-6.7), CD25 (PC61.5), B220 (RA3-6B2), CD19 (1D3), Ly6C (HK1.4), Ly6G (RB6-8C5), TNF-α (MP6-XT), interferon-gamma (IFN-γ) (XMG1.2), IL-10 (JES5-16E3), and IL-6 (MP5-20F3). For intracellular staining, cells were fixed and permeabilized with Foxp3 staining buffer (Thermo Scientific, Pittsburg, PA, USA). All antibodies were obtained from BD Biosciences (San Diego, CA, USA), Biolegend (San Diego, CA, USA), or eBioscience (San Diego, CA, USA). Additional information for all antibodies is listed in Table S2. Stained cells were acquired using FACSCalibur (BD Biosciences). Flow cytometry datasets were analyzed with the FCS Express software (version 5, De Novo Software, Glendale, CA, USA), and dead cells were excluded by forward and side scatter characteristics.

### 2.4. Cytokine Assays

TNF-α and IL-6 levels in cell culture media were quantified using ELISA kits (PeproTech, Rocky Hill, NJ, USA) and following manufacturer’s instructions.

### 2.5. Detection of hAAT

hAAT level in the culture medium was detected by hAAT specific ELISA, as previously described [52,53]. Briefly, a microtiter plate (Immulon 4, Dynex Technologies, Chantilly, VA, USA) was coated with goat anti-hAAT (Bethyl, Montgomery, TX, USA) in Voller’s buffer overnight at 4 °C. The plate was blocked with 3% BSA (Sigma-Aldrich, St. Louis, MO, USA) for 1 h at 37 °C. Duplicate standard curves (hAAT; Sigma-Aldrich, St. Louis, MO, USA) and samples were incubated at 37 °C for 1 h. Rabbit anti-hAAT (Sigma-Aldrich, St. Louis, MO, USA) was reacted with the captured antigen at 37 °C for 1 h. A third antibody, goat anti-rabbit IgG conjugated with peroxidase (Sigma-Aldrich, St. Louis, MO, USA) was incubated at 37 °C for 1 h. The plate was washed with PBS-Tween 20 (0.05%) between reactions. After adding substrate (O-Phenyldiamine, Sigma-Aldrich), the plate was read at 490 nm on an MRX microplate reader (DynexTechnologies, Chantilly, VA, USA).

hAAT in mouse tissues was detected by immunostaining, as previously described [54]. Briefly, rabbit anti-hAAT antibody (Fitzgerald Industries International, Actor, MA, USA) was used as primary antibody and goat anti-rabbit IgG conjugated with horseradish peroxidase (MACH 2 Rabbit HRP-Polymer, BIOCARE MEDICAL, Pacheco, CA, USA), which was used as a secondary antibody. Pictures were taken with 10× and 40× magnifications using the image analysis software (Aperio Imagescope v11.2.0.780, Aperio, Sausalito, CA, USA).

### 2.6. DAH and Lung Pathological Evaluation

DAH was evaluated by weight and gross inspection of excised lungs. The percentage of lung with hemorrhage was estimated in a blind manner where lung gross pathology was classified into no hemorrhage (0%), partial hemorrhage (25–75%), and complete hemorrhage (75–100%). Lung tissue samples were fixed overnight in 10% buffered formalin and embedded in paraffin. The embedded tissue was cut and stained with hematoxylin and eosin (H&E). 

### 2.7. Statistical Analysis

Graphing and statistical analysis were performed using GraphPad Prism (v.)504 (La Jolla, CA, USA). Differences between groups were compared using ANOVA with Tukey’s post hoc tests. Graphs show mean and SEM, and significance levels are presented as * *p* < 0.05, ** *p* < 0.01, and *** *p* < 0.001.

## 3. Results

### 3.1. Splenocytes from hAAT-Tg Mice Are Less Susceptible to Activation

**Figure 1:**
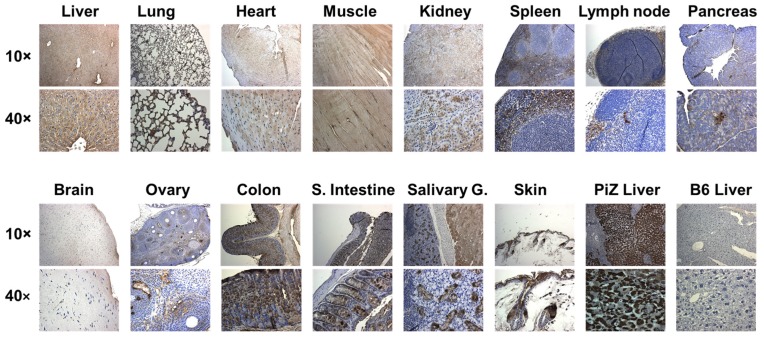
Immunostaining for hAAT expressed in tissues from hAAT-Tg mice. For each tissue, one 10× (top row) and one 40× (bottom row) images are presented. Human patient liver (PiZ Liver) was used as a positive control, showing a brown signal for hAAT specific staining, and C57BL/6 liver (B6 Liver) was used as a negative control. S. Intestine for small intestine and Salivary G. for salivary gland.

In previous studies, we showed that recombinant adeno-associated viral (rAAV) vector- expressed hAAT has a protective effect in spontaneous autoimmune disease models, including the NZM2410 lupus-prone mice [49]. To test the effect of transgenic hAAT protein in induced disease models, we generated a hAAT transgenic mouse line using the rAAV vector plasmid DNA [51]. These hAAT-Tg mice express high levels of hAAT, which can be detected in the circulation (4.5 ± 2.2 mg/mL, *n* = 12) [51] and in different tissues including the lung, heart, kidneys, liver, and spleen (Figure 1). In an additional experiment, we used tissues from B6 mice as controls to confirm that the detection signals are hAAT specific (Figure S1).

Immune cell activation plays an important role in lupus pathogenesis and may lead to DAH as a complication [2]. To test the effect of hAAT on immune cell activation, we compared splenocytes from B6 and hAAT-Tg mice with or without LAC for 6 h using flow cytometry. The frequency of total. 

**Figure 2:**
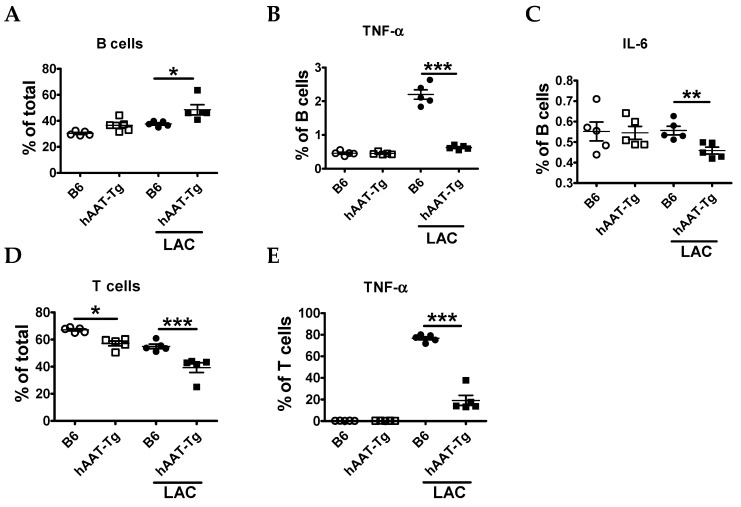
Effect of human alpha-1-antitrypsin (hAAT) on splenocytes from B6 and hAAT transgenic (hAAT-Tg) mice. Splenic B cells were identified as CD19^+^B220^+^ and T cells were identified as CD3^+^CD19^−^. (**A**) Frequency of B cells relative to total live splenocytes. Frequency of proinflammatory B cells producing TNF-α (**B**) and IL-6 (**C**). (**D**) Frequency of T cells gated on total live cells. (**E**) Frequency of proinflammatory T cells producing TNF-α. Data are presented as the mean ± SEM for five mice per group and analyzed by one-way ANOVA using Tukey’s post hoc test. Open circles are for cells from B6 mice. Open squares are for cells from hAAT-Tg mice. Filled circles are for cells from B6 mice and treated with LAC. Filled squares are for cells from hAAT-Tg and treated with LAC. * *p* < 0.05, ** *p* < 0.01, *** *p* < 0.001.

CD19^+^B220^+^ B cells was higher in hAAT-Tg than in B6 stimulated splenocytes (Figure 2A). However, the frequency of TNF-α and IL-6 producing B cells was lower in hAAT-Tg than in B6 splenocytes (Figure 2B,C). The frequency of CD3^+^CD19^−^ T cells was also lower in hAAT-Tg than in B6 splenocytes with and without LAC activation (Figure 2D). After LAC treatment, the frequency of TNF-α producing T cells was lower in AAT-Tg mice (Figure 2E). 

**Figure 3:**
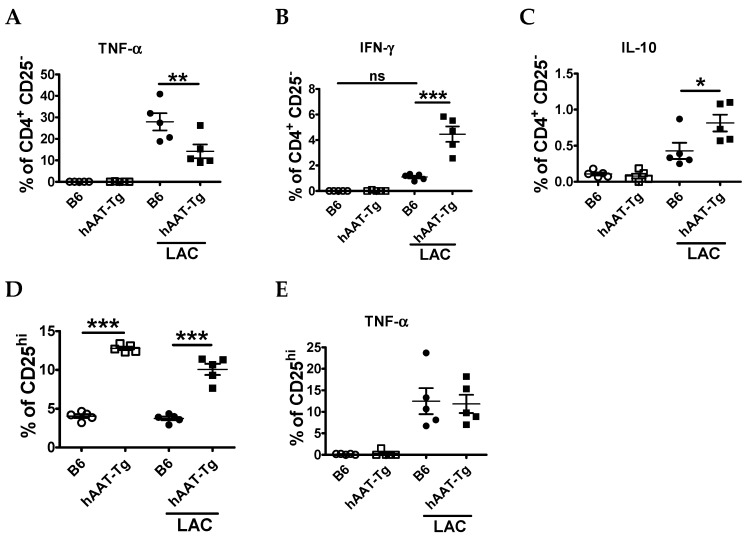
Effect of hAAT on splenic T cell populations. (**A**) Frequency of CD4^+^CD25^−^ cells producing TNF-α. (**B**) Frequency of CD4^+^CD25^−^ cells producing interferon-gamma (IFN-γ). (**C**) Frequency of CD4^+^CD25^−^ cells producing IL-10. (**D**) Frequency of CD25^hi^ T cells (gated on total CD4^+^ cells), and (**E**) Frequency of CD25^hi^ T cells producing TNF-α. Data are presented as the mean ± SEM for five mice per group and analyzed by one-way ANOVA using Tukey’s post hoc test. Open circles are for cells from B6 mice. Open squares are for cells from hAAT-Tg mice. Filled circles are for cells from B6 mice and treated with LAC. Filled squares are for cells from hAAT-Tg and treated with LAC. * *p* < 0.05, ** *p* < 0.01, *** *p* < 0.001.

We next characterized the effect of hAAT on T cell populations. The frequency of TNF-α producing CD4^+^ T cells was lower in hAAT-Tg than in B6 stimulated splenocytes (Figure 3A). Although the frequency of IFN-γ CD4^+^ T cells were higher in hAAT-Tg splenocytes (Figure 3B), the frequencies of IL-10 producing CD4^+^CD25^−^ T cells, as well as CD4^+^CD25^hi^ T cells, which include regulatory T cells (Tregs), were higher in hAAT-Tg mice (Figure 3C,D). In this setting, the frequency of TNF-α producing CD4^+^CD25^+^ cells was similar in both B6 and hAAT-Tg mice (Figure 3E).

**Figure 4:**
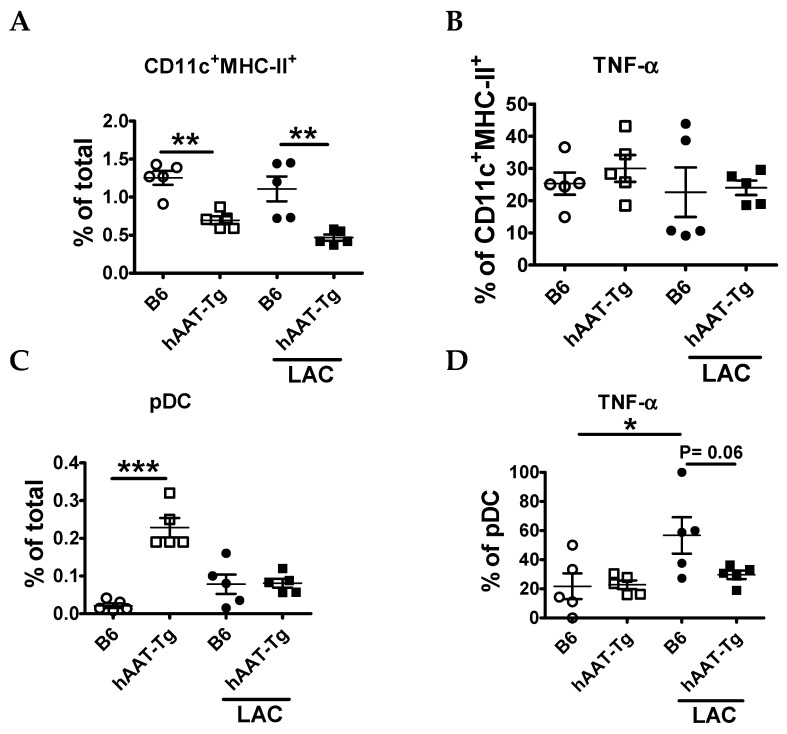
Effect of hAAT on splenic DCs from B6 and hAAT-Tg mice. Splenic conventional dendritic cells (cDCs) were identified as CD11C^+^MHC-II^+^ and plasmacytoid DCs (pDC) as PDCA-1^+^B220^+^CD11c^lo^. (**A**) Frequency of cDCs. (**B**) Frequency of cDCs producing TNF-α, (**C**) Frequency of pDCs, and (**D**) Frequency of pDCs producing TNF-α. Data are presented as the mean ± SEM for five mice per group and analyzed by one-way ANOVA using Tukey’s post hoc test. Open circles are for cells from B6 mice. Open squares are for cells from hAAT-Tg mice. Filled circles are for cells from B6 mice and treated with LAC. Filled squares are for cells from hAAT-Tg and treated with LAC. * *p* < 0.05, ** *p* < 0.01, *** *p* < 0.001.

The frequency of conventional dendritic cells (cDCs, CD11c^+^MHC-II^+^ DCs) was lower in hAAT-Tg mice with or without LAC treatment than in B6 mice (Figure 4A). LAC treatment did not induce TNF-α (Figure 4B) production or IL-6 in cDCs (data not shown), and there was no difference between strains. It is well known that plasmacytoid dendritic cells (pDCs) are the major source of IFN-I; however, they can also contribute to the production of other cytokines, such as TNF-α [55]. Although the frequency of pDCs was higher in hAAT-Tg than B6 mice (Figure 4C), the frequency of TNF-α producing pDCs was lower in hAAT-Tg mice (Figure 4D). Taken together, these results indicated that hAAT-Tg expression decreases activation of immune cells.

### 3.2. Transgenic Expression of hAAT Inhibited Splenocyte Activation by TLR4 and TLR7/8agonists

**Figure 5:**
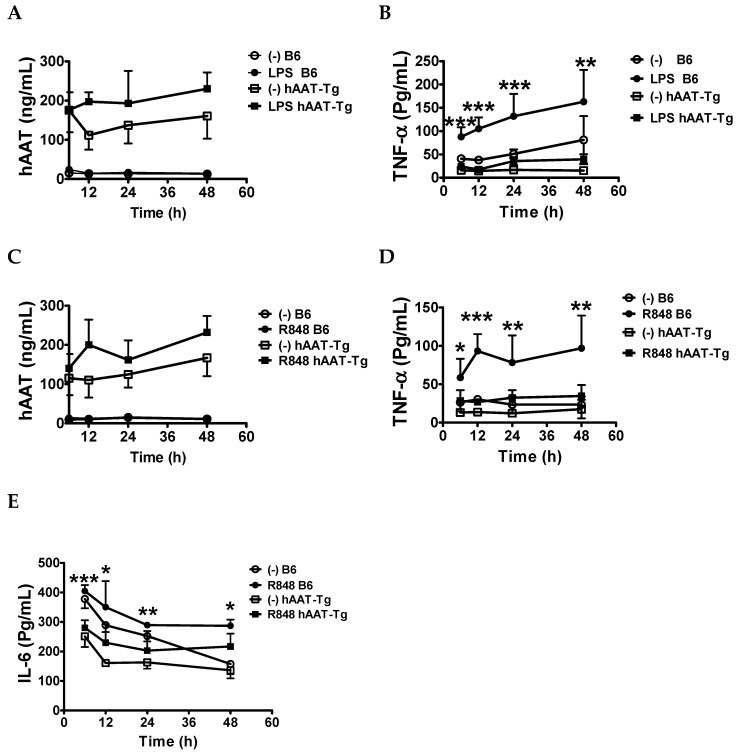
The inhibitory effect of hAAT in LPS or R848-treated splenocytes. Splenocytes from B6 and hAAT-Tg mice were treated with 10 µg/mL LPS, or 1 µg/mL R848, for 6, 12, 24, and 48 h. Supernatant was collected for hAAT and cytokine detection by ELISA. hAAT (**A**) and TNF-α levels (**B**) with or without LPS. hAAT (**C**), TNF-α (**D**), and IL-6 (**E**) levels with or without R848. Data are presented as the mean ± SEM for five mice per group and analyzed by one-way ANOVA using Tukey’s post hoc test. * *p* < 0.05, ** *p* < 0.01, *** *p* < 0.001.

Previously, we have shown that hAAT treatment inhibited BMDC activation and cytokine secretion upon stimulation with TLR4 or TLR9 agonists in B6 and lupus-prone B6. Sle1.Sle2.Sle3 (B6.TC) mice [48,49]. To further investigate the protective effect of hAAT on immune cell activation, splenocytes from B6 and hAAT-Tg mice were stimulated with or without 10 µg/mL of LPS or 1 µg/mL of R848 for up to 48 h. Consistent with the ubiquitous expression of hAAT in the Tg mice (Figure 1), hAAT was detected in the culture medium of LPS and R848 activated splenocytes from hAAT-Tg mice, and at higher levels than in non-activated hAAT-Tg splenocytes (Figure 5A,C). TNF-α levels were significantly lower in LPS-stimulated hAAT-Tg than B6 splenocytes (Figure 5B). Similarly, R848-stimulated TNF-α and IL-6 levels were significantly lower in hAAT-Tg than in B6 splenocytes (Figure 5D,E). These results are consistent with the results obtained with LAC activation of specific splenocyte populations (Figure 2, Figure 3 and Figure 4) and clearly demonstrated that hAAT has an inhibitory effect on proinflammatory cytokine productions. 

### 3.3. Transgenic Expression of hAAT Prevents Pristane Induced DAH

**Figure 6:**
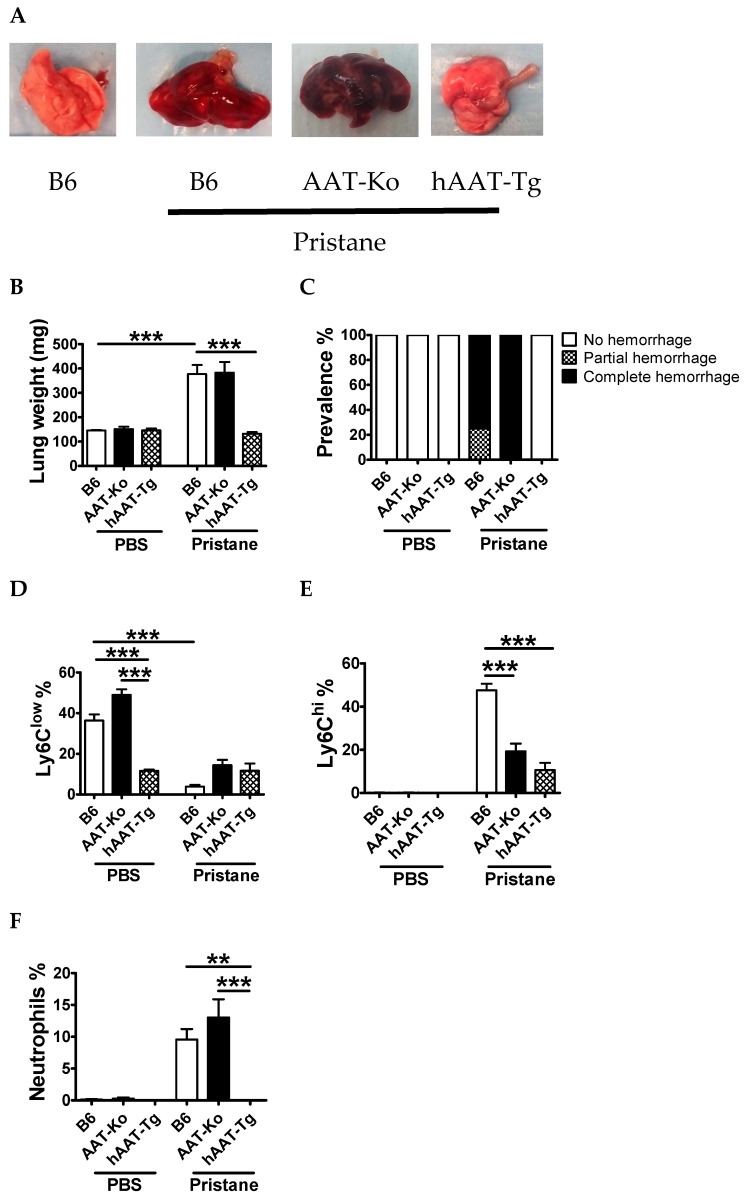
hAAT prevented pristane induced diffuse alveolar hemorrhage (DAH) in hAAT-Tg mice. B6, AAT-Ko, and hAAT-Tg mice were treated with or without pristane and sacrificed 7 days later. (**A**) Representative lung images. (**B**) Fresh lung weight. (**C**) Prevalence of DAH. Peritoneal exudate cells (PECs) from some of the animals were analyzed by flow cytometry. (**D**) Frequency of Ly6C^low^ cells (CD11b^+^Ly6C^low^). (**E**) Frequency of Ly6C^hi^ monocytes (CD11b^−^Ly6C^hi^). (**F**) Frequency of neutrophils (CD11b^+^Ly6G^+^ Ly6C^int^). For D, E, and F, B6 mice (pristane-treated *n* = 4, PBS *n* = 5), mouse AAT-Ko (pristane-treated *n* = 4, PBS *n* = 2), and hAAT-Tg (pristane-treated *n* = 5, PBS *n* = 3) mice were used. Data are presented as the mean ± SEM and analyzed by one-way ANOVA using Tukey’s post hoc test. ** *p* < 0.01, *** *p* < 0.001.

In order to investigate the protective effect of hAAT against DAH, we induced DAH in B6, hAAT-Tg, as well as AAT-Ko mice. One week after pristane or PBS injection, all animals were sacrificed. As shown in Figure 6A–C, the gross anatomy of lungs from hAAT-Tg mice was completely normal, whereas lungs from B6 and AAT-Ko mice showed partial to severe hemorrhage. To gain insights into mechanisms, peritoneal exudate cells (PECs) were analyzed by flow cytometry. Pristane treatment significantly reduced the frequency of Ly6C^low^ resident monocytes, but increased the frequency of Ly6C^hi^ induced inflammatory monocytes and neutrophils in B6 and AAT-Ko mice. In contrast, the frequency of Ly6C^low^ cells remained at the same level and the frequency of Ly6C^hi^ cells and neutrophils was lower in hAAT-Tg mice than in B6 mice (Figure 6D–F). 

### 3.4. Protective Effect of hAAT Protein Therapy against DAH

**Figure 7:**
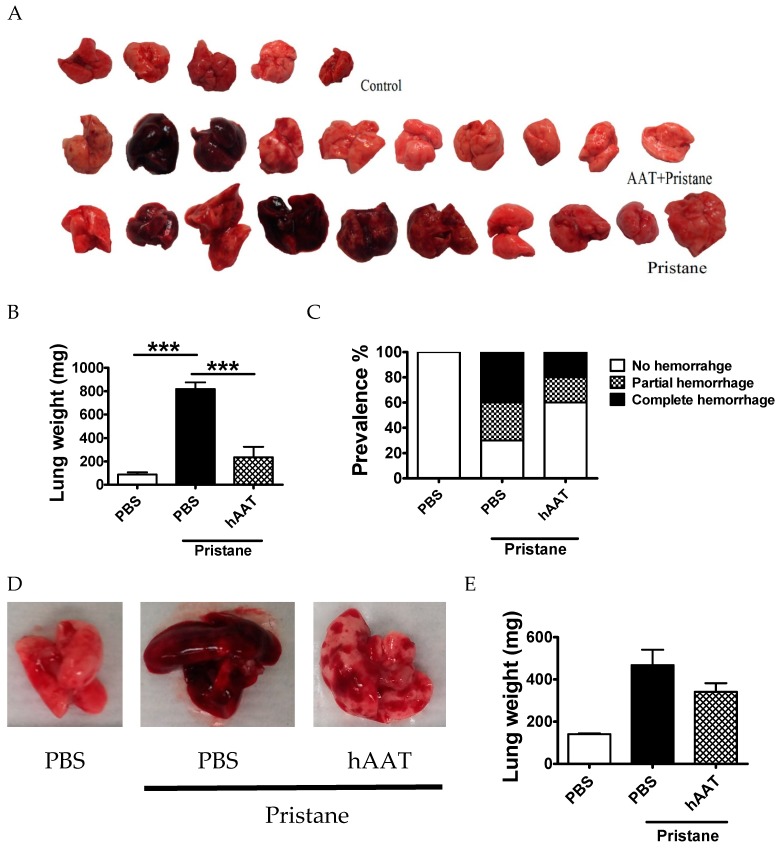

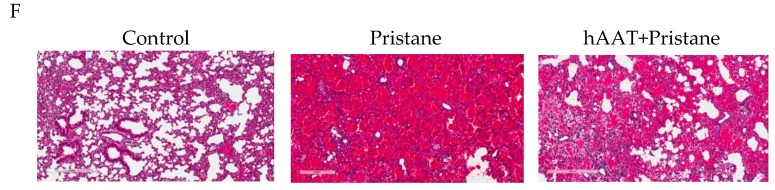
AAT protein therapy protected against pristane-induced DAH. B6 mice were divided into three groups, control group (*n* = 5), hAAT treated group (2 mg/IP/every 2 days, *n* = 10), and pristane treated group (*n* = 10). Mice in hAAT and pristane group received single IP pristane injection and lungs were examined 14 d later. (**A**) Lung gross pathology. (**B**) Fresh lung weight and (**C**) Prevalence of DAH. In a similar experiment, animals was treated with hAAT for 7 days and lungs were examined. (**D**) Lung gross pathology. (**E**) Fresh lung weight. (**F**) Representative H&E staining of lung from animals sacrificed 7 d after pristane injection. Data are presented as the mean ± SEM and analyzed by one-way ANOVA using Tukey’s post hoc test *** *p* < 0.001. The white bar represents 300 µm. IP: Intraperitoneal; H&E: hematoxylin and eosin.

To investigate the protective effect of hAAT treatment on pristane-induced DAH, B6 mice received hAAT (2 mg IP every 2 days) or PBS (100 µL IP every 2 days) for one week before the injection of pristane. Mice were then treated with hAAT for 2 more weeks and sacrificed. As shown in Figure 7, hAAT treatment significantly reduced the occurrence and severity of lung hemorrhage (Figure 7A–C). A second cohort of animals was sacrificed 7 d after pristane injection. Lung weight, severity of lung hemorrhage, and H&E staining of the lungs also showed reduced lung hemorrhage in hAAT treated group, although no statistical significance was achieved, likely due to small sample size and short treatment time (Figure 7D–F). Together, these results demonstrated that hAAT treatment has a protective effect against pristane-induced DAH.

## 4. Discussion

Results from this study indicate a new potential treatment option for DAH. DAH is one of the life-threatening complications in SLE patients. Because of the limitations (e.g., side effects and inefficiency) of current treatment options using steroids and immunosuppressant dugs, development of a safe and efficient therapy for DAH is needed. In the present study, we showed that hAAT Tg expression or treatment significantly prevented pristane-induced DAH in a mouse model and strongly indicated the therapeutic potential of hAAT for DAH. The protective effect of hAAT in the lungs has been well documented in AATD and COPD patients [44,56]. The therapeutic effect of hAAT was shown in several other disease models, including type 1 diabetes [53,57,58], arthritis [52,59], lupus [48,49], stroke [60], bone loss [43,61,62,63], and graft versus host disease (GVHD) [64,65]. However, the effect of hAAT on DAH has not been reported previously. Results from this study extend our current understanding of hAAT functions and applications. Since hAAT is a Food and Drug Administration (FDA) approved drug with an excellent safety profile [56], these results may be translated into a clinical application in humans.

Several mechanisms may contribute to the protective effect of hAAT against pristane-induced DAH. (1) Inhibition of neutrophil activation. Neutrophils play an important role in DAH pathogenesis [3,8] and hAAT inhibits neutrophil infiltration and function secretions [47,66]. Consistently, we showed that Tg expression of hAAT completely blocked pristane induced neutrophil recruitment. (2) Altering the balance of monocyte populations. There are two subpopulations of monocytes: anti-inflammatory (or resident) monocytes (Ly6C^low^) and inflammatory monocytes (Ly6C^hi^), which contribute significantly to DAH development [2,13]. Tg expression of hAAT completely blocked pristane-mediated reduction of Ly6C^low^ cells and inhibited pristane-mediated increase of Ly6C^hi^ cells. (3) Inhibition of DC activation and alteration of DC populations. Consistent with our previous observations in spontaneous lupus models [48,49], we showed that Tg hAAT lowered cDC frequency. Interestingly, the frequency of pDCs in hAAT-Tg mice was higher than that in B6 or AAT-Ko mice, which is consistent our previous observation that hAAT gene therapy increases the frequency of pDCs in NZM2410 mice [40]. We have reported that hAAT treatment increased the frequency of tolerogenic CCR9^+^ pDCs [48,49,67]. In addition, hAAT promotes the expansion of tolerogenic semimature DCs (smDC) [68], inhibits cDC and pDC activation and function in B6 and B6.TC mice [27,28]. (4) Inhibition of the expression of inflammatory cytokines. We showed that splenocytes, as well as B and T cells from hAAT-Tg mice, produce less IL-6 and TNF-α in response to stimulation. In addition, two other mechanisms may also contribute to the prevention of DAH. First, hAAT is a well-known proteinase inhibitor, which can inhibit a wide range of proteinases, such as neutrophil elastase. Since proteinases play an important role in tissue damage, the inhibition from hAAT may contribute to the protection [69]. Second, hAAT can interact with inflammatory mediators (e.g., IL-8, TNF-α receptors, and leukotriene B4) and block their actions in inflammation sites [47,70]. It is possible that all the above mechanisms support each other and work together for the protective effect [69]. For example, the production of proinflammatory cytokines and chemokines by B cells contribute to pristane-induced DAH by recruiting monocytes and neutrophils to the peritoneal cavity and lungs [3]. The recruitment of these inflammatory cells into lungs preceded hemorrhage by several days, indicating the pivotal role of these cells in the pathogenesis of pristane-induced DAH [10]. Nonetheless, the identification of the precise mechanisms underlying the protective effect of AAT against DAH will require further investigation.

As a member of the SERPIN superfamily, hAAT shares some common structural and functional features with other SERPINs, including viral SERPINs. They use their reactive center loop (RCL) to interact with serine proteinases and form an inactive complex with one-to-one molar ratio [71]. The target serine proteases of hAAT and viral SERPINs include neutrophil elastase, cathepsin G, proteinase 3, and other enzymes, which are major factors in pathogenesis of inflammation and tissue damage [72]. Although these SERPINs have diverse functions, many of them play important roles in immune regulation [27]. In the present study, we showed that hAAT treatment can protect against pristane-induced DAH. It is possible that other members of the SERPIN superfamily may have a similar protective effect. For example, it has been shown that viral SERPIN (SERP1) can reduce unrelated viral-induced lung hemorrhage [37]. Future studies using SERP1 or other viral SERPINs in the pristane-induced DAH mouse model may provide a better understanding of the protection mechanism and suggest more therapeutic options for DAH.

It should be noted that pristane-induced DAH mouse model has some limitations. First, while in humans DAH is life-threatening, most of the mice survive and recover from pristane-induced DAH [3]. In the work presented here, all animals survived to the end of the experiments. Second, the immune system in mice is different from that in humans, although they are similar. These factors should be considered in future translational studies. 

## 5. Conclusions

Collectively, we have shown that hAAT inhibited proinflammatory cytokine producing cells induced regulatory cells, and inhibited TLR4 and TLR7/8 stimulation. Human AAT prevented pristane induced DAH in hAAT-Tg mice and hAAT treated mice and reduced peritoneal Ly6C^hi^ monocytes and neutrophils. 

## Supplementary Materials

The following are available online at https://www.mdpi.com/2077-0383/8/9/1341/s1, Figure S1: Expression of hAAT in tissues from hAAT-Tg mice. Representative immunostaining images (10×) of from B6 (top, as negative controls) and hAAT-Tg mice are presented. Brown color in the tissue section indicates hAAT-positive signals. The white bar represents 300 µm, Table S1: Groups of animals in each experiment, Table S2: List of antibodies used for flow cytometry.
