# Supplementary material for: Alpha-1-Antitrypsin Ameliorates Pristane Induced Diffuse Alveolar Hemorrhage in Mice
Source: J Clin Med. 2019 Aug 29;8(9):1341. doi: 10.3390/jcm8091341 (PMC6780888; doi:10.3390/jcm8091341)
Supplement: Supplementary file 1 [file jcm-08-01341-s001.pdf]

Journal of
Clinical Medicine

MDPI

Article

Alpha-1-Antitrypsin Ameliorates Pristane Induced Diffuse Alveolar Hemorrhage in Mice

Supplemental Materials

Table S1. Groups of animals in each experiment.

Studies	Strains	Age (Weeks)	N	Treatment	Days	Observations
I (Figure 2–5)	B6	8–12	5	Animals without any treatment were sacrificed		Characterizations of B cells, T cells and DCs; Secretion of hAAT, TNF-α, and IL-6 from splenocytes in response to LPS or R848.
	Tg		5			
II (Figure 6)	B6	10–12	5	PBS	7	Effect of transgenic expression of hAAT on pristane induced DAH
	B6		4	Pristane		
	Ko		2	PBS		
	Ko		4	Pristane		
	Tg		3	PBS		
	Tg		5	Pristane		
III (Figure 7A–C)	B6	8–10	5	PBS	14	Effect of hAAT protein therapy on pristane induced DAH
	B6		10	Pristane +PBS		
	B6		10	Pristane + hAAT		
IV (Figure 7D–F)	B6	10–12	5	PBS	7	Short term treatment of hAAT on pristane induced DAH
	B6		4	Pristane +PBS		
	B6		4	Pristane +hAAT		

Table S2. List of antibodies used for flow cytometry.

Binding Targets	Clone	Company	Catalog Number
CD16/CD32	93	eBiosciences	14-0161-85
CD11b	M1/70	eBioscience	11-0112-82
CD11c	HL3	BD Biosciences	550261
PDCA-1	927	Biolegend	652410
I-A/I-E	M5/114.15.2	BioLegend	107608
CD4	RM4-5	Biolegend	100516
CD3	145-2C11	BioLegend	100311
CD8	53-6.7	BioLegend	100722
CD25	PC61.5	eBioscience	25-0251-82
B220	RA3-6B2	BD Biosciences	553090
CD19	1D3	BD Biosciences	557399
Ly6C	HK1.4	Biolegend	128031
Ly6G	RB6-8C5	eBioscience	17-5931-82
TNF-α	MP6-XT	eBioscience	25-7321-82
IFN-γ	XMG1.2	BioLegend	505818

IL-10	JES5-16E3	BioLegend	505026
IL-6	MP5-20F3	BD Biosciences	554401

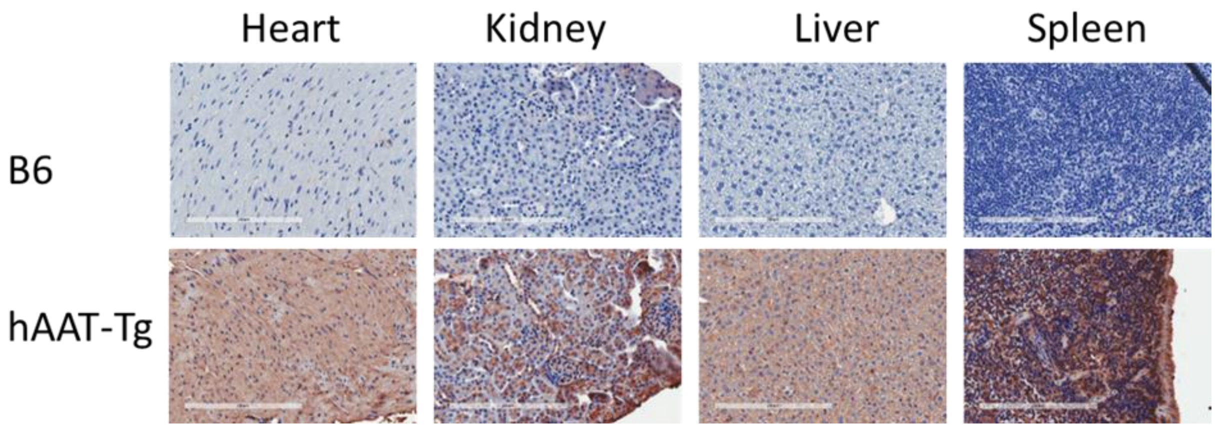

Figure S1. Expression of hAAT in tissues from hAAT-Tg mice. Representative immunostaining images (10×) of from B6 (top, as negative controls) and hAAT-Tg mice are presented. Brown color in the tissue section indicates hAAT-positive signals. The white bar represents 300 μm.
